# MoS_2_ Photoelectrodes for Hydrogen Production: Tuning the S-Vacancy Content in Highly Homogeneous Ultrathin Nanocrystals

**DOI:** 10.1021/acsami.3c02192

**Published:** 2023-07-05

**Authors:** Nuria Jiménez-Arévalo, Jinan H. Al Shuhaib, Rodrigo Bautista Pacheco, Dario Marchiani, Mahmoud M. Saad Abdelnabi, Riccardo Frisenda, Marco Sbroscia, Maria Grazia Betti, Carlo Mariani, Yolanda Manzanares-Negro, Cristina Gómez Navarro, Antonio J. Martínez-Galera, José Ramón Ares, Isabel J. Ferrer, Fabrice Leardini

**Affiliations:** † Departamento de Física de Materiales, Universidad Autónoma de Madrid, 28049, Madrid, Spain; ‡ Dipartimento di Física, Sapienza Università di Roma, 00185 Roma, Italy; § Physics Department, Faculty of Science, Ain Shams University, 11566 Cairo, Egypt; ∥ Departamento de Física de la Materia Condensada, Universidad Autónoma de Madrid, 28049 Madrid, Spain; ⊥ Instituto Nicolás Cabrera, Universidad Autónoma de Madrid, 28049 Madrid, Spain

**Keywords:** Molybdenum Disulfide, Electrocatalysis, Water Splitting, Defect Engineering, Sulfur Vacancies, Salt-Assisted Chemical Vapor Deposition

## Abstract

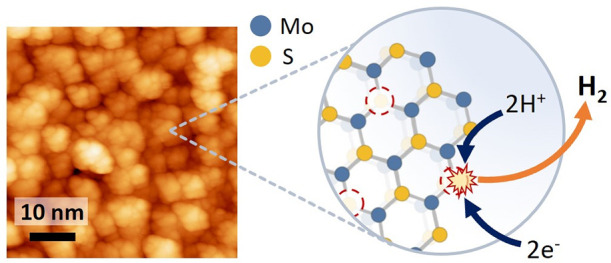

Tuning the electrocatalytic properties of MoS_2_ layers can be achieved through different paths, such as reducing their thickness, creating edges in the MoS_2_ flakes, and introducing S-vacancies. We combine these three approaches by growing MoS_2_ electrodes by using a special salt-assisted chemical vapor deposition (CVD) method. This procedure allows the growth of ultrathin MoS_2_ nanocrystals (1–3 layers thick and a few nanometers wide), as evidenced by atomic force microscopy and scanning tunneling microscopy. This morphology of the MoS_2_ layers at the nanoscale induces some specific features in the Raman and photoluminescence spectra compared to exfoliated or microcrystalline MoS_2_ layers. Moreover, the S-vacancy content in the layers can be tuned during CVD growth by using Ar/H_2_ mixtures as a carrier gas. Detailed optical microtransmittance and microreflectance spectroscopies, micro-Raman, and X-ray photoelectron spectroscopy measurements with sub-millimeter spatial resolution show that the obtained samples present an excellent homogeneity over areas in the cm^2^ range. The electrochemical and photoelectrochemical properties of these MoS_2_ layers were investigated using electrodes with relatively large areas (0.8 cm^2^). The prepared MoS_2_ cathodes show outstanding Faradaic efficiencies as well as long-term stability in acidic solutions. In addition, we demonstrate that there is an optimal number of S-vacancies to improve the electrochemical and photoelectrochemical performances of MoS_2_.

## 1 Introduction

The rational design of advanced electrocatalysts for green hydrogen production using water and electrical energy supply from renewable sources is a critical issue that must be addressed to reduce CO_2_ emissions and promote a transition to cleaner energies. In the past decade, many efforts have been made to optimize the performances of the cathodes and the anodes of the water electrolytic cells.^1,2^ In these electrodes, the hydrogen evolution reaction (HER) and the oxygen evolution reaction (OER) take place, which are the semireactions involved in the water electrolysis process. In particular, one of the most investigated electrocatalysts as a cathode for electrolytic water splitting during the last years is MoS_2_.^3−5^ This compound is a nontoxic material that can be grown in a 2D morphology and consists of relatively abundant elements.

There are several strategies to improve the electrocatalytic performance of MoS_2_ for the HER. A first approach consists of decreasing the number of layers in the films down to monolayers.^6^ This correlation between the number of layers and the electrochemical properties is related to electron hopping transport through the different layers of MoS_2_. Reducing the number of layers reduces the potential barriers that exist in the vertical plane of the samples, thus enhancing the charge transfer and increasing the efficiency. In addition, it has been reported that the edges of MoS_2_ flakes are the catalytically active sites for the HER, as the edges might serve as easier paths for transferring the hopping electrons than the basal plane, which remains relatively inert.^7,8^ Thus, several works have pursued different methodologies to increase the number of exposed edge sites by nanostructuring MoS_2_.^5^ Another approach to enhance the performance of MoS_2_ is by modifying its basal plane and making it more active through the transformation from a 2H to the metastable 1T phase, which has a higher conductivity and a more active basal plane.^9−11^ Finally, it has also been shown that introducing sulfur vacancies by different techniques (electrochemical, electron irradiation, or mild Ar plasma, among others) introduce gap states that favor hydrogen adsorption, therefore improving the electrocatalytic activity of MoS_2_ layers.^12−16^

In this work, we have designed a method to obtain highly defective MoS_2_ layers to be used as cathodes for the HER. For this purpose, we used a special salt-assisted chemical vapor deposition (CVD) method that allows using a lower growth temperature and high heating and cooling rates, thus reducing the growth time. In this way we can obtain films composed of ultrathin MoS_2_ nanocrystals, reducing the number of layers and increasing the number of edges. We also modified the basal plane of our nanocrystals by introducing a H_2_ flow during the CVD growth, which permits tuning the number of S-vacancies in the MoS_2_ layers.

To achieve a viable application of all of these materials for water splitting, the area of the electrodes must be considerable. As far as we know, only a few works have managed to create samples in the cm^2^ range.^6,17^ The present growth method allows the acquisition of highly homogeneous MoS_2_ layers over relatively large areas (cm^2^). We show a detailed analysis of the structural, optical, and chemical homogeneity of our samples, based on different characterization techniques. This strategy of nanostructuring and defect engineering opens up the possibility of tuning the properties of MoS_2_ electrodes by varying growth conditions and keeping good homogeneity control over large-area electrodes.

## 2 Experimental Techniques

### 2.1 MoS_2_ Growth

MoS_2_ was grown by salt-assisted chemical vapor deposition (CVD) on different substrates using a tubular quartz reactor with a 20 mm inner diameter. The precursors were heated by a cylindrical furnace mounted on two rails, which can be moved along the quartz reactor (see a diagram of the experimental process in Figure S1a).

The molybdenum oxide (MoO_3_, Sigma Aldrich, >99.5% purity) precursor was mixed with 20–25 wt % of NaCl (Scharlau, synthesis grade, >99% purity) to decrease its melting point.^18^ This method allows the growth of MoS_2_ layers at temperatures lower than those of the usual CVD method (see Figure S1c). About 4 mg of the MoO_3_ + NaCl mixture is placed in the center of an alumina crucible below the desired substrate. Another crucible containing sulfur powder as a chalcogen source was placed upstream 16 cm apart. In this way, a temperature gradient of 400 °C is created between the two precursors during CVD growth.

The electrical furnace was preheated at 600 °C and then moved to place the crucible with the substrate and the MoO_3_ precursor at its center (at 600 °C), while the sulfur (Merck, 99.99% purity) crucible was kept at 200 °C. After 15 min, the reactor was moved away and the system cooled naturally to room temperature. The furnace displacement allowed reaching high heating and cooling rates during growth (Figure S1b).

The syntheses were carried out under different Ar/H_2_ mixtures. The Ar flow was fixed at 150 sccm, and the H_2_ flow varied between 0 and 60 sccm.

Several substrates were used to grow MoS_2_ layers, such as fused silica slides (SPI Supplies), Si wafers, Si wafers covered with a 290 nm thick SiO_2_ layer (Si/SiO_2_, MicroChemicals), glassy-carbon disks (GC, Micro to Nano), and highly oriented pyrolytic graphite (HOPG, SPI Supplies). Silicon and carbon substrates were cleaned by using acetone and ethanol, whereas silica and Si/SiO_2_ substrates were further cleaned by oxygen plasma. As for the HOPG substrates, both sides were exfoliated using Scotch tape, thus creating fresh surfaces.

### 2.2 Characterization Techniques

The topography of the samples was studied with a homemade atomic force microscope (AFM) controlled by WSxM Software.^19^ The topography images were taken in dynamic, noncontact mode under ambient conditions with a commercial silicon AFM tip with a constant force *k* = 40 nN/nm and a resonance frequency of 300–350 kHz. Before that, the sample was gently scanned in contact mode to whip possible dirt accumulation due to the ambient conditions.

Scanning tunneling microscopy (STM) characterization was performed in an ultrahigh-vacuum (UHV) chamber with a base pressure in the 10^–10^ mbar range. This UHV system is equipped with a home-built variable temperature scanning tunneling microscope (VT-STM).^20^ STM measurements were performed with the bias voltage applied to the sample while the tip was grounded. STM data acquisition and analysis were executed by using the WSxM software.^19^ In this UHV system, the chemical characterization of the samples by means of Auger electron spectroscopy (AES) measurements was carried out by using a four-grid analyzer.

Information about the chemical composition of the samples was acquired by X-ray photoelectron spectroscopy (XPS) measurements, carried out in an ultrahigh-vacuum chamber with a base pressure in the low 10^–10^ mbar range. The XPS measurements were carried out at the SmartLab departmental laboratory of the Department of Physics at Sapienza University. The X-rays were generated by an Al Kα (1486.6 eV) monochromatic source (SPECS XR50 MF) with focused beam, and the photoelectrons were analyzed by a SPECS PHOIBOS 150 with an energy resolution of ∼0.4 eV and a spatial resolution better than 100 μm. Calibration of the binding energy (BE) position with respect to the Fermi level for the observed lines was done by acquiring the Au 4f_7/2_ (84.0 eV BE) core level after each measurement.

Raman and photoluminescence (PL) spectra were recorded using a confocal optical microscope with different lenses (20× and 100×), with a WiTec ALPHA 300AR instrument. The laser power was 0.1 mW, and the excitation wavelength was 532.3 nm.

Optical characterizations were done by using different setups. Macroscopic transmittance spectra (spot size of about 12 mm^2^) in the UV–vis–near-IR range were recorded on a Perkin-Elmer Lambda 1050 spectrophotometer. Microscopic transmittance and reflectance spectra (size of about 2 × 10^–3^ mm^2^) were recorded using a confocal optical microscope coupled to a CCD spectrometer.^21^

The electrochemical characterization was performed using a three-electrode photoelectrochemical cell. The reference electrode (RE) was a saturated calomel electrode (SCE) with a potential of *E*°_SCE_ = 0.248 V vs RHE. For the counter electrode (CE) we used a 9 cm^2^ platinum foil and the MoS_2_ grown on glassy carbon was placed as the working electrode (WE), with an apparent area of 0.79 cm^2^. Those three electrodes were immersed in a 0.5 M H_2_SO_4_ (pH = 0.3) aqueous solution and connected to a PGSTAT302N potentiostat–galvanostat (Autolab) provided with an integrated impedance FRA II module. In addition, an Ar flow of 20 sccm bubbled through the electrolyte during the experiment. The gases evolved were collected and driven to a mass spectrometer. Figure S2 shows a diagram of the photoelectrochemical cell employed in this work.

The measured electrode potentials (*E*_SCE_) have been converted to the reversible hydrogen electrode (*E*_RHE_) scale by using eqs 1 and 2.
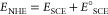
1
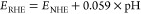
2

To characterize the photoresponse of our MoS_2_ samples, the WE was illuminated with a halogen lamp (Osram 650 W) so that the intensity reaching the surface sample was 65 W/m^2^.

## 3 Results

### 3.1 Growth of Homogeneous Nanocrystalline MoS_2_ Ultrathin Layers

**Figure 1:**
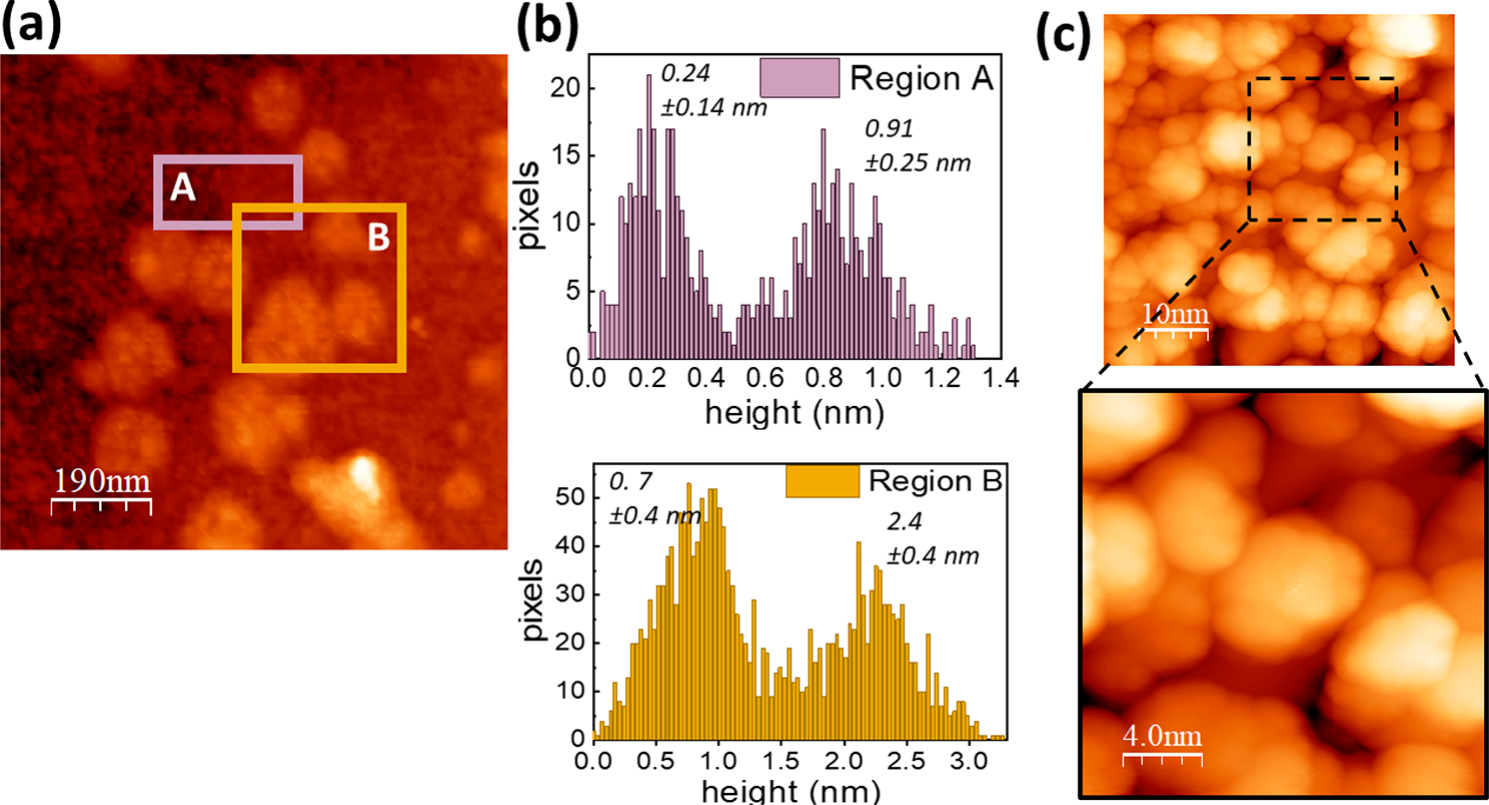
(a) AFM image of a MoS_2_ layer grown on Si/SiO_2_ substrate. (b) Topography distribution histograms obtained from the regions A and B. Values included in the figures indicate the mean values and standard deviations of each peak of the bimodal distribution. (c) STM image (50 × 50 nm^2^) of a MoS_2_ layer grown on HOPG and enlargement of a 20 × 20 nm^2^ region in the same zone. Tunneling parameters: *V*_s_ = −2.5 V and *I*_T_ = 20 pA.

The electrocatalytic properties of 2D materials can be enhanced by nanostructuring and defect engineering.^2,4,22^ Therefore, we aimed at growing highly defective MoS_2_ layers to investigate their use as cathodes for the HER. Salt-assisted CVD growth was selected, as it allows a lower growth temperature, which produces layers with low crystallite size and high defect density. The morphology of the obtained MoS_2_ layers was characterized by using different microscopies. A representative AFM image of MoS_2_ grown on Si/SiO_2_ is shown in Figure 1a. This image shows a nonhomogeneous film with two distinctive regions, A and B, with different contrasts hinting at a different thickness at each zone (detailed images in these two regions are shown in Figure S3). The average thickness in region A was 0.7 ± 0.3 nm and that in region B was 2.4 ± 0.6 nm, as deduced by the topography distribution histograms (Figure 1b). This result proves that MoS_2_ films are composed by 1–3 layer-thick crystals. This observation was further confirmed by STM imaging. The apparent height of the MoS_2_ crystals was obtained by analyzing line profiles in different regions of long-scale morphology images (see Figure S4). More interestingly, we could obtain short-scale STM images (Figure 1c), which evidenced the nanostructured morphology of our samples, that were composed by mono-, bi-, and trilayer nanocrystals of lateral dimensions of a few nanometers.

**Figure 2:**
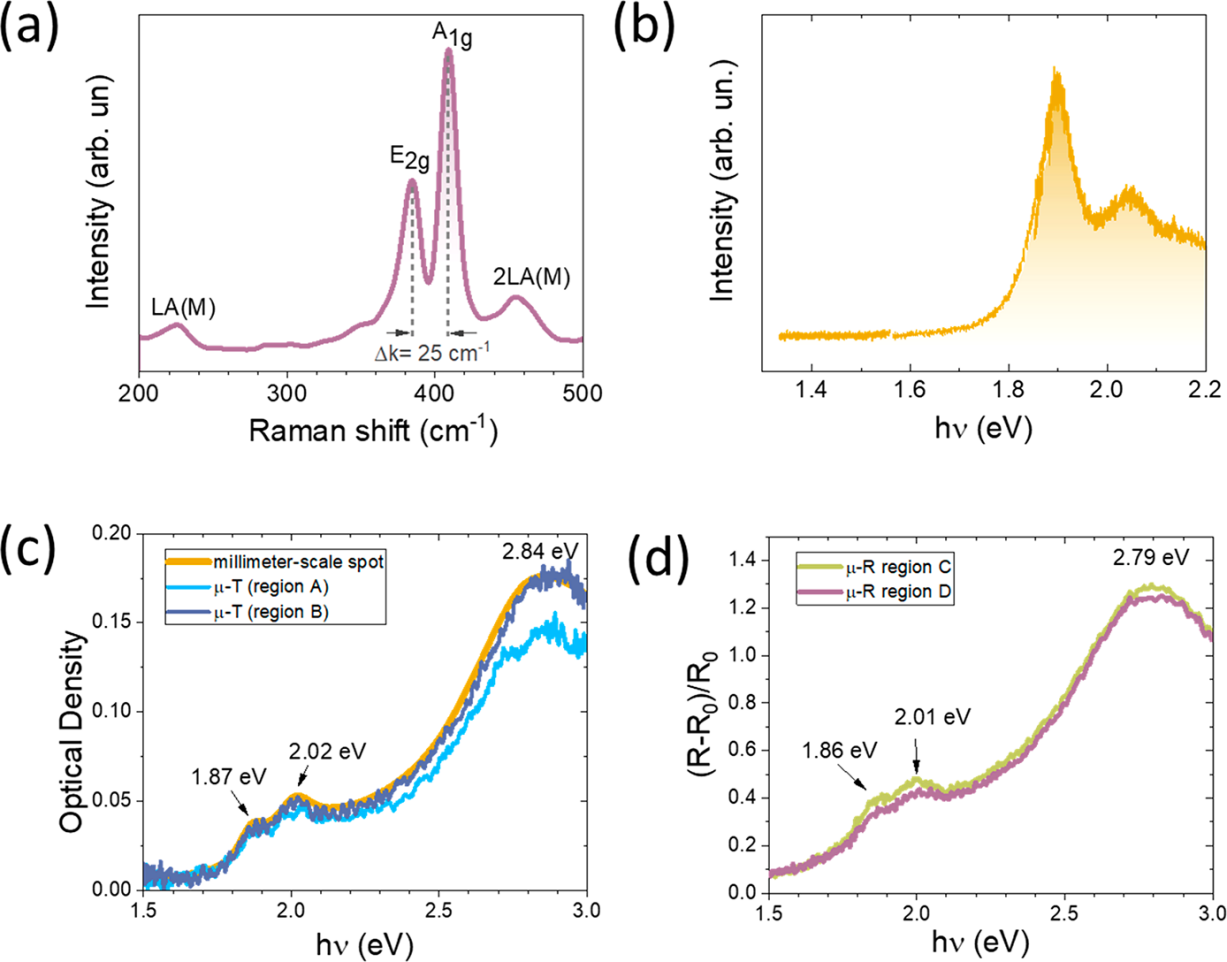
Optical characterizations of a MoS_2_ layer grown on different substrates: Si/SiO_2_ (a, b) and fused silica (c, d). (a) Raman spectrum and (b) PL spectrum. (c) Optical density measurements done with a macroscopic setup (millimeter-scale spot size) and with a microtransmittance setup (micrometer-scale spot size) recorded in two different zones of the sample. (d) Differential reflectance measurements acquired with a microreflectance setup (micrometer-scale spot size) in two different zones of the sample, to show the homogeneity in the optical properties of the films.

Figure 2a shows the characteristic Raman spectrum of a typical MoS_2_ sample grown on fused silica. The two main Raman bands of MoS_2_ were observed at ∼384 and ∼409 cm^–1^.^9^ It must be noted that the difference of the wavenumber shift of these two bands (Δ*k* = A_1g_ – E_2g_) is equal to 25 cm^–1^. This value does not match those reported in the literature for monolayer, bilayer, or trilayer MoS_2_ flakes, which are around 20.5, 22.4, and 23 cm^–1^, respectively.^6,23^ However, it coincides with that reported for MoS_2_ nanocrystals, which is about 25 cm^–1^.^24^ Some authors have suggested that larger Δ*k* values may be related to smaller crystalline domains.^23^ This result agrees with the nanostructured morphology of our samples observed by STM. On the other hand, two additional Raman peaks appear at 227 and 454 cm^–1^, respectively. The first band corresponds to the LA(M) Raman mode of MoS_2_ that has been also observed in MoS_2_ nanoparticles^24^ and is related to structural defects.^25^ The band centered at 454 cm^–1^ corresponds to the second order of LA(M) (2LA(M)) and to A_2u_ at 468 cm^–1^.^26,27^

The in-plane nanometric morphology of MoS_2_ also shows a fingerprint in the PL spectrum. Figure 2b shows the PL spectrum of a MoS_2_ layer grown onto Si/SiO_2_. The maximum of the PL peak appears at 1.90 eV (653 nm), at higher energy than that observed for micrometric-size flakes, namely 1.83 eV (677 nm).^18,28^ It has been reported that the PL spectrum of nanosized MoS_2_ is blue-shifted compared to micrometric-scale flakes.^29,30^ Therefore, the PL spectrum of the present layers also indicates the nanometric size of the MoS_2_ flakes.

A critical aspect of CVD growth is the large-scale homogeneity of the obtained layers. A first indication of the spatial homogeneity of the layers can be obtained by measuring their optical properties. The optical density of a MoS_2_ layer grown on SiO_2_ was recorded with a macroscopic setup and a microtransmittance setup in different zones of the sample a few millimeters apart from each other. Results from both measurements coincide quite well, which is a good indication of sample homogeneity. In a rough approximation, the layer thickness was obtained from our optical density results and the reported absorption coefficient, whose maximum value was 7.5 × 10^5^ cm^–1^.^28^ Given that the maximum optical density value in our samples was 0.175, an overall thickness of 2.1 nm is obtained. Considering that the MoS_2_ layer grew on both sides of the fused silica substrate, the thickness corresponds to an average value of 1.6 layers on each side, in good agreement with the AFM and STM results. This good homogeneity of the MoS_2_ layers and the average thickness were further confirmed by recording differential reflectance spectra with micrometric-size resolution at different zones of the sample, as shown in Figure 2d. Indeed, the peak position of the optical absorption peaks^31^ and the differential reflectance peaks^21^ in MoS_2_ layers depend on their thickness. The peak positions in the transmittance and differential reflectance peaks of the present layers (see Figure 2d) are between those reported for monolayer and trilayer MoS_2_,^21,31^ thus confirming our previous results on the thickness obtained by AFM, STM, and optical density measurements. Moreover, owing to the relationship among the differential reflectance, the optical density, and the real part of the refractive index (*n*),^32^ we have obtained *n* of the MoS_2_ layers from the optical absorption and differential reflectance spectra experimentally measured with the present samples. The results are shown in Figure S5. The features observed in the dispersion curve of the refractive index (which exhibits saddle points for photon energies close to those of the absorption peaks) explain why the peak positions of the transmittance and reflectance peaks are slightly different (Figure 2c,d).

**Figure 3:**
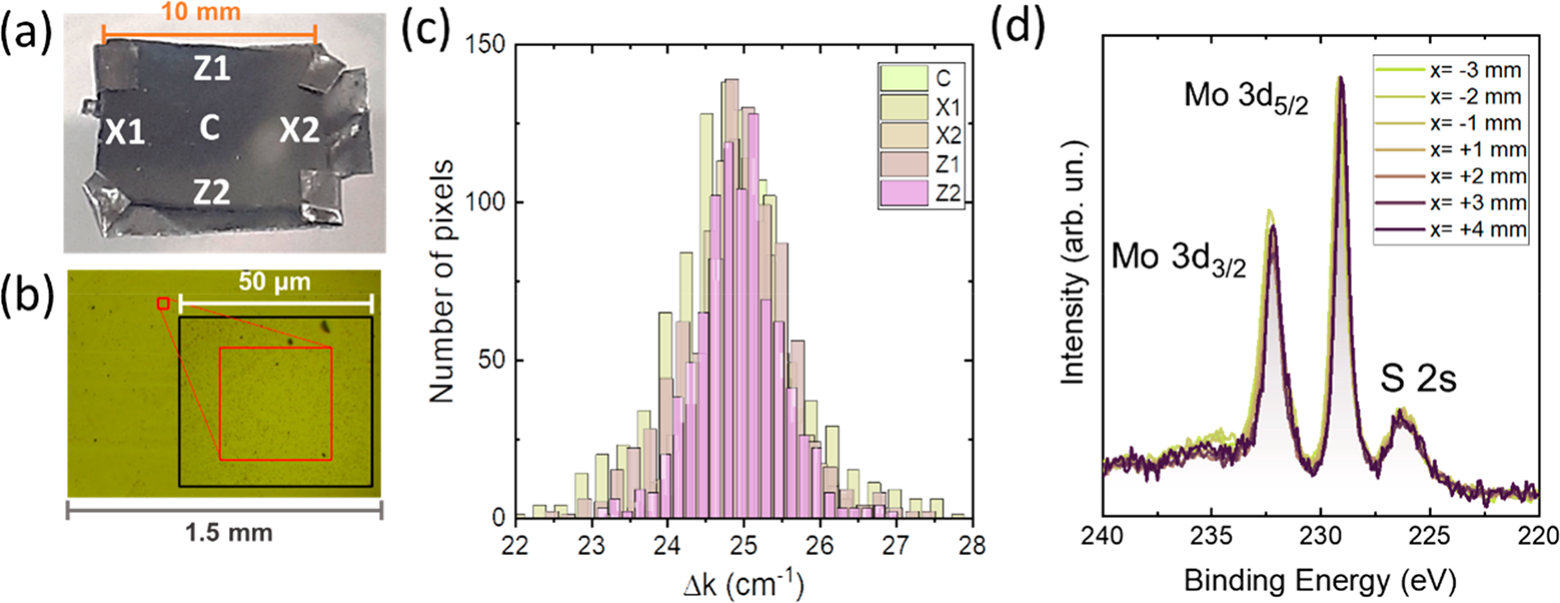
(a) Picture of the MoS_2_ sample grown on Si and mounted on a tantalum sample holder used for XPS measurements. Positions of the zones where Raman mappings were acquired are labeled. (b) Optical microscopy image. The red square shows the region C in (a) in which the Raman mapping was performed. (c) Histogram distributions of Δ*k* values in the Raman spectra for the five regions indicated in (a). (d) Micro-XPS spectra recorded at different zones of a line scan of 7 mm in length along the *X* axis. Mo 3d and S 2s BE peaks are shown.

The homogeneity of the structural properties of the MoS_2_ layers was further investigated by micro-Raman spectroscopy measurements at different points of a MoS_2_ sample grown on Si (Figure 3). The sample was oriented in the CVD reactor with the *X* direction indicated in Figure 3a parallel to the Ar flow. To compare the Raman spectra in different zones of the sample, five Raman mappings of 900 spectra each were obtained in regions of 30 × 30 μm^2^ (Figure 3b) at different positions of the sample (indicated in Figure 3a). Histogram distributions of the difference in A_1g_ and E_2g_ peak positions (Δ*k*) have been obtained for each map, and the results are shown in Figure 3c. We consider that each one of the 900 spectra acquired in a mapping is a “pixel”. The relative intensities between A_1g_ and E_2g_ Raman modes were also recorded and are represented in Figure S6. These histograms show no significant variation in the Raman spectra acquired at different zones. This is a clear indication of the good spatial homogeneity of the present samples.

The spatial homogeneity in the chemical bonding state and the stoichiometry were investigated by micro-XPS measurements (with a spot size in the 100 μm range) in two different MoS_2_ samples grown on Si. Line scans with about 7–9 points with a step of 1 mm were acquired in both samples along two perpendicular directions for each one. Figure 3d shows the XPS spectra in seven different regions in a line of 7 mm along the *X* axis (additional spectra can be seen in Figure S7). Spectra were background subtracted and normalized to the most intense peak for a better comparison. Mo 3d and S 2s peaks were selected to characterize the chemical bonding state and the stoichiometry. As can be observed, the variation in the peak position and the relative intensities are negligible, confirming the chemical homogeneity along the sample. More details about the XPS results are given in section 3.2.

The results of the different characterization techniques presented in this section clearly indicate that the obtained MoS_2_ layers have a thickness of between 1 and 3 layers and are formed by nanocrystals with in-plane lateral dimensions of a few nanometers. This morphology is related to the salt-assisted CVD method used here, which allowed us to grow the present MoS_2_ layers at 600 °C using fast heating and cooling rates (Figure S1b). This growth temperature is considerably lower than that typically used, namely 750 °C. The low growth temperature and the high heating and cooling rates used here as compared to previous literature works are the main reasons we obtained a nanocrystalline morphology. On the other hand, the salt-assisted synthesis method is based on the creation of intermediate MoO_*x*_Cl_*y*_ species that favor the evaporation of Mo precursors at lower temperatures.^18^ Some previous works have reported the contamination of their samples with Na^33,34^ when using the salt-assisted CVD method. Nevertheless, we did not observe any Cl or Na contamination (as can be seen in the XPS survey spectra shown in Figure S8). This could be because we use lower temperatures in our CVD (600 °C) than in those previous reports (650 °C^33^ or 740 °C^34^). The use of a low growth temperature favors the creation of nanometric-size crystals over large areas, with no contaminants from the NaCl precursor. This fact confers to our layers some specific features in the Raman and PL spectra compared to exfoliated or microcrystalline MoS_2_ layers. In addition, this morphology gives rise to a high density of edges along the samples. Finally, our MoS_2_ layers present excellent spatial homogeneity over areas in the cm^2^ range. As far as we know, this is the first experimental report showing spatial homogeneity of chemical bonding and related properties for ultrathin nanocrystals, while homogeneous samples have been previously obtained only in micrometer-sized crystalline MoS_2_ flakes.^23^

### 3.2 Tuning the Sulfur Content in the MoS_2_ Layers

**Figure 4:**
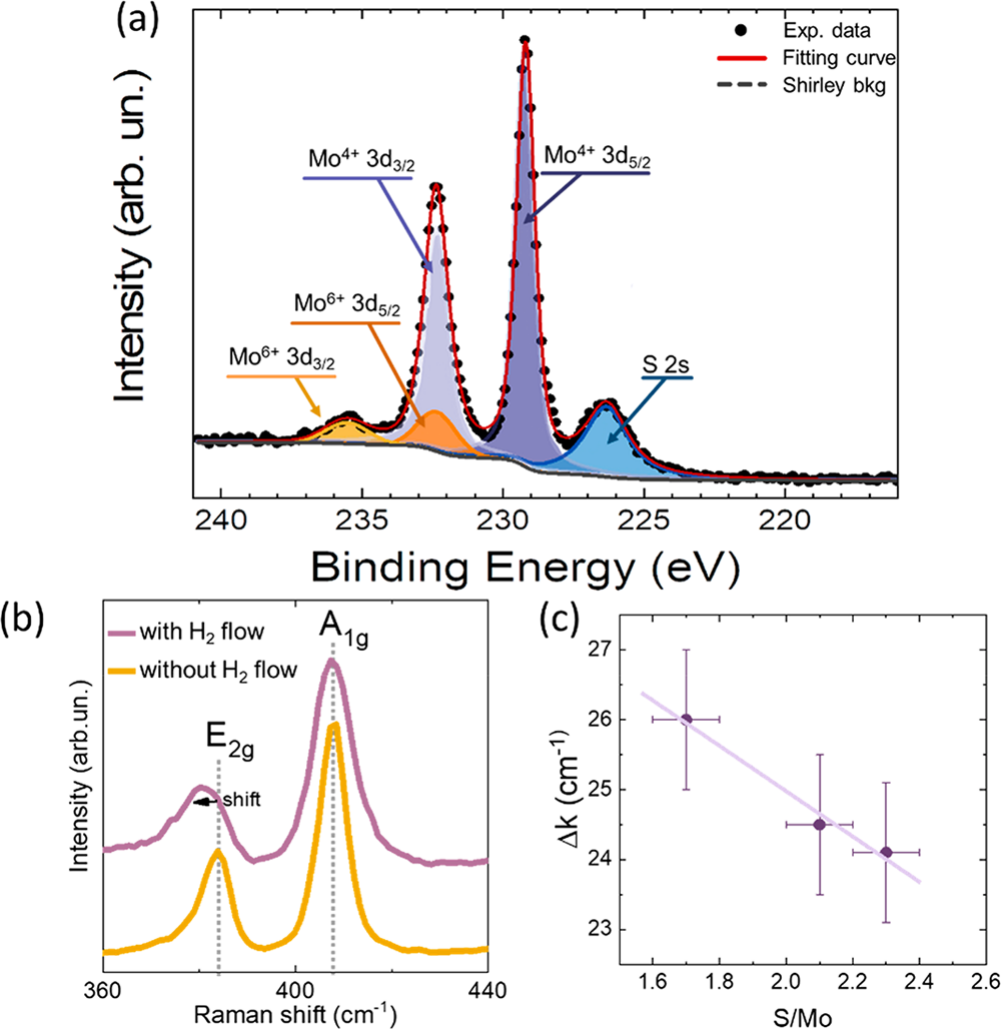
(a) XPS spectrum of a MoS_2_ sample (MS-1.7) in the region of Mo 3d, obtained at a pass energy of 5 eV: experimental data (dots), single-component fitting curves (colored and filled lines), complete fitting curve (red continuous line), and Shirley-shape background (gray line). (b) Raman spectra for samples grown with and without the use of a hydrogen flow during the salt-assisted CVD. (c) Raman features (Δ*k* values) against stoichiometry (S/Mo ratio) for three different samples grown under different conditions.

To tune the properties of the obtained MoS_2_ layers, in particular, the electrocatalytic activity for the HER, we varied growth conditions. Specifically, it has been previously shown that the presence of an H_2_ flow during CVD growth may favor the creation of sulfur vacancies in MoS_2_.^35,36^ Therefore, three MoS_2_ samples were grown under different Ar/H_2_ mixtures. Afterward, the sulfur content was determined by XPS. Figure 4a shows a representative XPS spectrum of the Mo 3d and S 2s core levels for a MoS_2_ layer grown under an Ar/H_2_ mixture of 150/30 sccm. The curves were analyzed and fitted using pseudo-Voigt line shapes (Lorentzian–Gaussian curves), after subtracting a Shirley-shape background as a fitting parameter. We could observe five peaks (positions in BE are indicated in Table 1) that correspond to the Mo^6+^ 3d_3/2_ and 3d_5/2_, due to the presence of MoO_3_, and Mo^4+^ 3d_3/2_, 3d_5/2_, and S 2s, ascribed to MoS_2_.^37−41^ Spectra were recorded in more than 15 spots of our samples at different positions (section 3.1), and the average value for the positions in the BE for the three samples is shown in Table 1. Similar BE values have been observed for these samples, and their values are in agreement with previous results (Table 1 and references therein).

**Table 1:** Binding Energies for Five Components of the XPS Spectra Shown in Figure 4a Compared to Those in the Literature^a^.

	binding energy (eV)					
name/ref	S^2–^ 2s	Mo^4+^ 3d_5/2_	Mo^4+^ 3d_3/2_	Mo^6+^ 3d_5/2_	Mo^6+^ 3d_3/2_	S 2s/Mo 3d
MS-2.3^e^	226.6 ± 0.3	229.4 ± 0.2	232.6 ± 0.3	232.4 ± 0.2	235.2 ± 0.2	2.3 ± 0.1^b^
MS-2.1^e^	226.5 ± 0.1	229.4 ± 0.1	232.5 ± 0.1	232.6 ± 0.2	235.7 ± 0.2	2.1 ± 0.1^b^
MS-1.7^e^	226.5 ± 0.2	229.4 ± 0.1	232.6 ± 0.3	232.6 ± 0.3	235.4 ± 0.2	1.7 ± 0.1^b^
(37)	227.04	229.76	232.82	not resolved	not resolved	2.33^b^
						2.06^c^
(38)	226.3	229.1	232.3	not resolved	not resolved	2.09^c^
(39)	∼227	229.7	232.9	231.5	234.7	2.3^d^
(40)	226.3	229.1	232.2	not resolved	235.9	not reported
(41)	226.3	229.1	232.2	not resolved	235.5	not reported

a The S/Mo ratio is also indicated. Samples are labeled according to their S/Mo ratio. The S 2p and Mo 3p components were also measured and are shown in Figure S9 and Table S1.

b Value obtained from S 2s/ Mo 3d peaks.

c Value obtained from S 2p/Mo 3d peaks.

d XPS peaks from which this ratio was obtained were not indicated.

e This work.

The stoichiometry of the samples was calculated by fitting the relative peak intensities of the S 2s and the Mo^4+^ 3d core levels, by taking into account the electron ionization cross-sections,^42^ and the S/Mo ratios are shown in Table 1. The normalized S/Mo intensity ratio decreases from 2.3 to 1.7 with an increase in the H_2_ flow used in CVD growth. Therefore, our XPS analyses suggest that the stoichiometry of the MoS_2_ layers can be easily tuned by using an H_2_ flow during CVD growth, favoring the creation of sulfur vacancies.^35,36^ In some of our samples, we obtain an overstoichiometric sulfur content, probably associated with the fact that the edges of the present MoS_2_ nanocrystals end in sulfur atoms. However, we cannot discard the possible contamination of the surface of the MoS_2_ with an excess of elemental sulfur or the presence of Mo vacancies, since S/Mo values higher than 2 have also been reported in MoS_2_ micrometric-sized flakes^37−39^ (see Table 1).

The influence of the H_2_ flow used in CVD on the morphology of the MoS_2_ nanocrystals has been investigated by STM imaging on samples grown onto HOPG with and without the use of a H_2_ flow (Figure S10). It has been observed that the size of the MoS_2_ nanocrystals is reduced when a H_2_ flow is used. This fact can be related to a local cooling effect induced by the higher gas flow (150 sccm Ar + 30 sccm H_2_ versus 150 sccm Ar), which leads to the growth of smaller particles. Another possibility is that the H_2_ flow etches the borders of the MoS_2_ nanocrystals, thus diminishing their size.

On the other hand, the use of an H_2_ flow during CVD growth also has a fingerprint in the Raman spectra, by shifting the position of the E_2g_ Raman band (see Figure 4b). A clear relationship between the S/Mo stoichiometry and Δ*k* emerges. Raman mappings, like those shown in Figure 3, were done to get the average Δ*k* values with samples having different S/Mo ratios (Figure S11). From these results, Δ*k* values can be plotted against S/Mo ratios, as shown in Figure 4c. The lower the S/Mo ratio (i.e., higher S-vacancy content), the higher the Δ*k* value, as previously observed by other authors.^36,43^ This fact can be used to determine the S/Mo stoichiometry by measuring the Raman spectra and can be of interest when dealing with samples grown on electrically insulating substrates or those that are too large and do not fit into a conventional XPS sample holder. In fact, we have used this relationship to obtain the sulfur-vacancy content in the electrodes employed (deposited on GC conductive substrates) for water electrolysis by measuring their Raman spectra (see section 3.3). However, we cannot discard the possible influence of the size of the MoS_2_ nanocrystals on the separation of the Raman bands. We have observed that increasing the H_2_ flow decreases not only the S/Mo ratios but also the size of the MoS_2_ nanocrystals, and both effects produce a separation of the Raman peak positions. In any case, we consider that there is a correlation between all these three parameters (S/Mo content, nanocrystal size, and separation of the Raman bands), thus the use of the Raman peak separation to estimate the S/Mo content in our electrodes (see section 3.3) is valid.

### 3.3 Use of MoS_2_ Layers as Electrocatalysts for the Hydrogen Evolution Reaction

The electrocatalytic activity of our ultrathin nanocrystalline MoS_2_ samples for the HER was investigated using different analytical methods. To this aim, three samples with different S contents were grown on glassy-carbon (GC) substrates. We used the linear relationship previously obtained between the sulfur content and the Raman shift differences (Figure 4c) to determine the stoichiometry from Raman measurements. The samples were labeled MS-2.2, MS-1.8, and MS-1.6, referring to the S/Mo ratio of each sample (Figure S12 shows their corresponding Δ*k* histograms).

**Figure 5:**
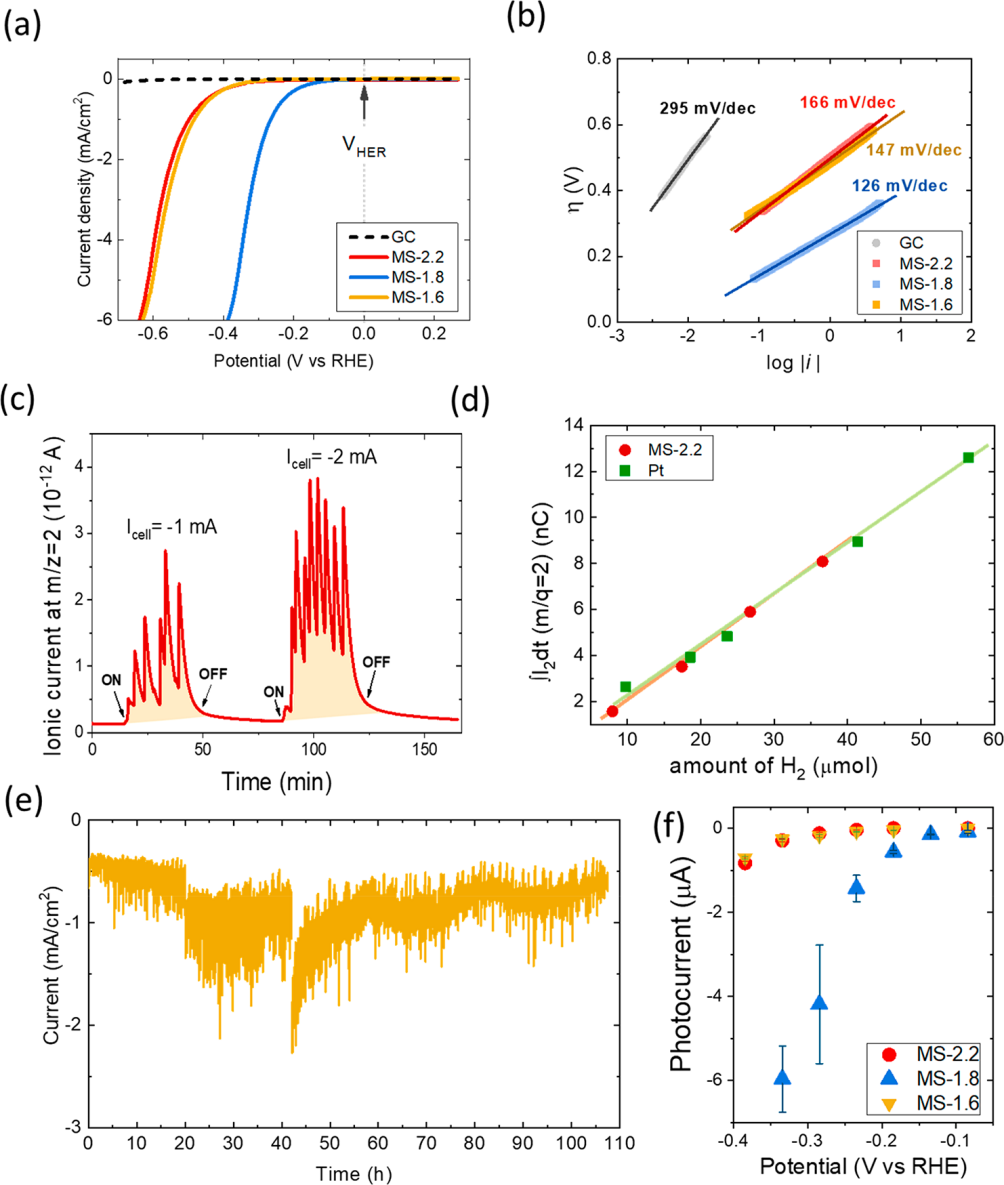
(a) Polarization LSV curves of a bare GC and of MoS_2_ samples with different S/Mo ratios (2.2, 1.8, and 1.6). The vertical dotted line indicates the equilibrium onset potential of the HER. (b) Tafel plots for the same samples and their corresponding linear fits. (c) Time evolution of the *i*_2_ mass spectrometric signal recorded at two applied electrolytic currents for sample MS-2.2. (d) Time integrals of *i*_2_ signals as a function of the theoretical amount of H_2_ generated at the electrodes (obtained by using the Faraday law). (e) Chronoamperometry test recorded at a bias potential of −0.484 V vs RHE for sample MS-1.6. (f) Photocurrents of the MoS_2_ samples as a function of the applied electrode potential.

Figure 5a shows the linear sweep voltammetry (LSV) curves of the three MoS_2_ samples as well as the curve recorded with a bare GC electrode. The GC presents weak current densities and poor electrocatalytic activity for the HER compared with the MoS_2_ samples. Therefore, the current densities recorded with our layers must be caused by the MoS_2_ layers and not by the GC substrates. Besides, the MS-1.8 sample provided the best response for the HER, showing the lowest overpotential for the HER of the three samples. On the other hand, samples MS-2.2 and MS-1.6 show similar electrocatalytic activity despite having different S/Mo ratios. A possible explanation of the observed differences in the electrocatalytic activities of the samples could be related to the different morphologies of the obtained MoS_2_ nanocrystals when the H_2_ flow in CVD growth was varied (see section 3.2). Therefore, electrochemically active surface areas have been determined by double-layer capacitance measurements for samples obtained under different growth conditions, and the results are shown in Figure S13. It can be seen that the surface areas of the samples are very similar and, therefore, cannot account for the observed differences in the electrocatalytic activities. Further details about the obtention of electrochemically active surface areas are given at the Supporting Information. Moving away from these considerations, we also investigated the possibility of Pt contamination on our electrodes (since we used Pt CE in our electrochemical experiments) that could influence our electrochemical data.^44^ Based on the results of the different characterizations (see more details in Figure S13), we concluded that there is not a significant effect of Pt leaching and deposition on our electrochemical results.

To determine the electrocatalytic activity of our samples, a Tafel analysis (Figure 5b) was performed, as it helps to determine the reaction mechanism that is taking place by the value of the Tafel slope. The slope values were 295, 166, 126, and 147 mV/dec for GC, MS-2.2, MS-1.8, and MS-1.6 electrodes, respectively (the values of the fitting parameters are given in Table S2). Tafel slopes are related to the overpotential. So, the lower the slope, the lower the potential increment necessary to increase the current density in one decade. It can be seen that MS-1.8 presents the lower Tafel slope among all the samples. This Tafel slope value indicates that the rate-limiting step for the HER could be a Volmer step or a Herovsky step with high coverage of hydrogen atoms on the surface of the electrodes, as is discussed in ref (45). A comparison of our values with those already reported in the literature is not straightforward. As was discussed by Shinagawa et al.,^45^ there is a dependence of the Tafel slope on the potential range. In many cases, Tafel slopes are calculated in a range of low overpotentials (and thus low electrolytic current densities), as lower Tafel slopes are observed in that range. However, this can be nonrepresentative of the electrochemical behavior of the electrodes. In our case, we have obtained Tafel slopes in the range of overpotentials and current densities representative of the LSV curves shown in Figure 5a. Few articles have analyzed potential ranges similar to ours and obtained values comparable to those we are obtaining.^6,11,17^ In particular, Yu et al.^6^ grew MoS_2_ layers on GC substrates with the same areas as ours and studied the layer dependence on the electrocatalytic properties. Their best Tafel plot value was 140 mV/dec for the one-layer MoS_2_ cathode. Comparing that result with ours, the positive effect of nanostructuring MoS_2_ and creating S-vacancies can be observed, as our best sample presents lower values of the Tafel slope.

Faradaic efficiencies of the MoS_2_ cathodes were determined by quantitative mass spectrometric analyses of the gases that evolved during the electrolysis. Further details on the experimental setup for gas analysis and calibration measurements done for quantitative analysis can be found in the Supporting Information of ref (46). The time evolution of the *i*_2_ mass spectrometric current (which is proportional to the H_2_ flow evolved from the MoS_2_ cathode) for sample MS-2.2 recorded for two fixed applied electrolytic currents is shown in Figure 5c. This signal presents some peaks due to the formation of bubbles at the surface of the MoS_2_ cathodes. Hydrogen gas produced at the surface builds up to make large bubbles before detaching from the electrode (see more details in the Supporting Information of ref (46)). By integrating the area under the mass spectrometric curves (after background subtraction), we can obtain a quantity proportional to the amount of hydrogen produced during these tests. These quantities can be plotted against the theoretical values calculated by using the Faraday law (i.e., by considering the total Coulombic charge that passes through the electrodes by applying a constant current during a fixed time). By plotting the results of the MS-2.2 sample together with those obtained in a similar experiment with a Pt foil (Figure 5d), it can be observed that the Faradaic efficiency of our samples is comparable to that of Pt, namely, close to 100%, as our data match perfectly with those obtained for Pt. This result indicates that secondary reactions, such as other possible redox processes of the MoS_2_ cathodes, are negligible.

Long-term stability tests are also a critical factor for practical applications. To test this facet in our samples, we conducted chronoamperometry measurements at −0.484 V vs RHE for more than 100 h (doing measurements at time intervals of about 20 h), as is shown in Figure 5e. The recorded signal appears to be noisy because H_2_ bubbles cover part of the electrode surface; therefore, the apparent area decreases (thus decreasing the electrolytic current) until the bubbles detach and the absolute value of the current increases. Nevertheless, the present electrochemical tests show that our MoS_2_ cathodes present excellent stability over long periods. In fact, Raman analyses performed after the electrochemical tests are similar to those recorded before (Figure S14). This is a good indication of the durability of our MoS_2_ cathodes.

Finally, we investigated the photoelectrochemical response of the MoS_2_ samples. As far as we know, there have been just a few works dealing with the photocatalytic and photoelectrocatalytic properties of MoS_2_ for hydrogen production, and most of them used combinations of MoS_2_ and other materials.^47−51^ Only one previous work unsuccessfully attempted to measure the photoelectrochemical properties of MoS_2_ in acidic H_2_SO_4_ media.^52^ In our case, to investigate the photoresponse of our MoS_2_ nanocrystals, we illuminated the MoS_2_ cathodes with a halogen light source and measured the photocurrents, i.e., the increase in the electrochemical current during the illumination (see Figure S15). Photocurrents were recorded at different electrode potentials ranging between 0 and −0.4 V versus RHE, as shown in Figure 5f. Measurements were not recorded for applied potentials below −0.4 V vs RHE, due to noise caused by bubble depletion. As can be observed, the photocurrents are negative, evidencing the p-type behavior of our electrodes. In addition, the lower the applied electrode potential, the higher the net photocurrent, a behavior usually presented in many semiconductors.^53^ Furthermore, the sample MS-1.8 displays the highest photocurrents, indicating a relationship between the electrocatalytic properties and the photoresponse. The photoresponses of the MS-2.2 and MS-1.6 samples are pretty similar, in agreement with their comparable electrocatalytic behavior.

The best electro- and photoelectrocatalytic performance has been obtained with sample MS-1.8. This result suggests a relationship between the S/Mo ratio and the electrochemical performance in which there is an optimal stoichiometry. There is a previous work showing that creating S-vacancies in the basal plane of a 2H-MoS_2_ monolayer introduces gap states that improve the electrocatalytic properties for the HER. In that work, S-vacancies were created by Ar plasma treatments of MoS_2_ monolayers with micrometric-sized crystalline domains. In particular, it was demonstrated that to increase the electrocatalytic performance of MoS_2_ there is an optimal number of S-vacancies.^15^ This same behavior was obtained by other researchers, who varied the number of S-vacancies by chemical etching and, thus, the activity of MoS_2_.^54^ Therefore, our investigation suggests that the relationship between the S-concentration (S/Mo ratio) and the electrochemical performance is universal, independent of the size of the MoS_2_ crystals (micrometric or nanometric) and the method used to generate these vacancies. In addition, we show that the S-concentration also affects the photoelectrochemical response of the MoS_2_ layers. As far as we know, this is the first report showing a relationship between the stoichiometry of MoS_2_ and photoelectrochemical properties in MoS_2_.

To summarize, all these results point out the excellent electro- and photoelectrocatalytic properties of the nanocrystalline MoS_2_ samples for the HER. We emphasize the high homogeneity over relatively large areas of our electrodes, an excellent outcome with respect to traditional electrode sizes, opening a pathway to use MoS_2_ electrodes in real electrolyzers.

## 4 Conclusions

Ultrathin nanocrystals of MoS_2,_ prepared using a salt-assisted CVD method, with variable S contents, were investigated as cathodes for hydrogen production by electrolytic water splitting. A successful strategy to obtain ultrathin nanocrystals with a high density of edges was achieved by decreasing the sublimation temperature thanks to the addition of NaCl to the MoO_3_ precursor, favoring the creation of 1–3-layer-thick nanocrystals with in-plane dimensions of a few nm, as determined by AFM and STM. In turn, this growth procedure promotes very high structurally and chemically homogeneous layers, with specific features of the Raman and the PL spectra (namely, a separation of Raman bands and a blue shift of the PL emission as compared to micrometric-sized flakes). Furthermore, the S/Mo ratio in the layers can be tuned by introducing a H_2_ flow during the CVD growth, as deduced from the XPS core level spectra and the relationship between the E_2g_ and A_1g_ Raman shift difference. Finally, the (photo)electrochemical response of large-area MoS_2_ electrodes (0.8 cm^2^) was analyzed in a 3-electrode electrochemical cell. Faradaic efficiencies close to 100% were determined by mass spectrometric analyses of the H_2_ that evolved during electrolysis. In addition, our samples presented an outstanding stability during long-term chronoamperometry tests (times over 100 h). Finally, our results demonstrate that the electrochemical and photoelectrochemical responses of MoS_2_ can be optimized by tuning the stoichiometry and finely controlling the growth conditions. This work opens new possibilities to scale up and tune MoS_2_ electrocatalytic properties by nanostructuring and defect engineering by varying growth conditions.

## Supporting Information Available

Schematic diagram and heating ramp of the growth and relative mass loss of the precursors, schematic diagram of the photoelectrochemical cell used, AFM images, STM image, apparent height, and Auger spectrum, refractive index of the MoS_2_ samples, histogram of the relative intensity between the A_1g_ and E_2g_ Raman bands, XPS spatial line scans in the Mo 3d binding energy region, XPS survey spectra, binding energies for the S 2p and Mo 3p components of the XPS spectra compared to those in the literature, XPS spectra in the S 2p and Mo 3p binding energy regions, STM images on MoS_2_ samples grown under different conditions, histogram distributions of the Δ*k* values in the Raman spectra for samples with different S/Mo ratios, values of the fitting parameters obtained from the Tafel plots, determination of the electrochemical surface active area from CV scans recorded at different scan rates with MoS_2_ electrodes grown under different conditions, Raman spectra before and after the electrochemical measurements, and chronoamperometry measurements under dark and illumination conditions (PDF)
