# Supplementary material for: MoS2 Photoelectrodes for Hydrogen Production: Tuning the S-Vacancy Content in Highly Homogeneous Ultrathin Nanocrystals
Source: ACS Appl Mater Interfaces. 2023 Jul 5;15(28):33514–24. doi: 10.1021/acsami.3c02192 (PMC10865293; doi:10.1021/acsami.3c02192)
Supplement: Supplementary file 1 — am3c02192_si_001.pdf [file am3c02192_si_001.pdf]

Supporting Information

MoS_2 photo-electrodes for hydrogen production: tuning the S-vacancies content in highly homogeneous ultrathin nanocrystals.

Nuria Jiménez-Arévalo[1], Jinan H. Al Shuhaib[1], Rodrigo Bautista Pacheco[1], Dario Marchiani[2], Mahmoud M. Saad Abdelnabi[2,3], Riccardo Frisenda[2], Marco Sbroscia[2], Maria Grazia Betti[2], Carlo Mariani[2], Yolanda Manzanares-Negro[4], Cristina Gómez Navarro[4,5], Antonio J. Martínez-Galera[1,5], José Ramón Ares[1], Isabel J. Ferrer[1,5], Fabrice Leardini[1,5]*

[1] *Departamento de Física de Materiales, Universidad Autónoma de Madrid, 28049, Madrid, Spain*
[2] *Dipartimento di Física, Sapienza Università di Roma, 00185, Roma, Italy*
[3] *Physics Department, Faculty of Science, Ain Shams University, 11566,Cairo, Egypt*
[4] *Departamento de Física de la Materia Condensada, Universidad Autónoma de Madrid, 28049, Madrid, Spain.*
[5] *Instituto Nicolas Cabrera, Universidad Autónoma de Madrid, 28049, Madrid, Spain*
* Corresponding author: fabrice.leardini@uam.es

Experimental Techniques

MoS_2 growth

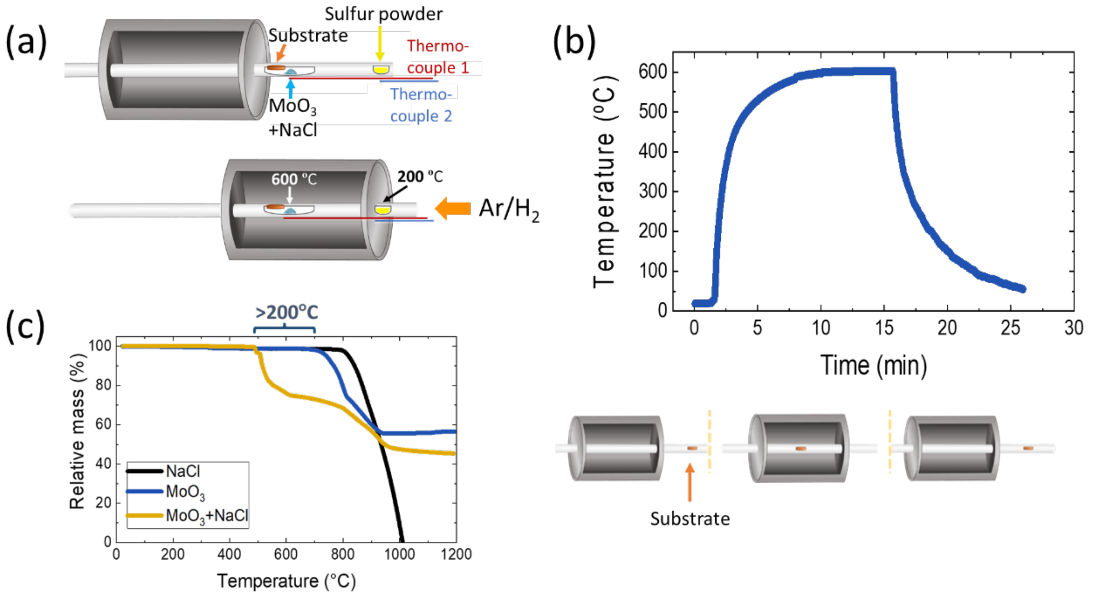

Figure S1. (a) Schematic diagram of the growth procedure to synthesise the MoS_2 samples with the salt-assisted CVD method. (b) Temperature profile used for CVD growth, indicating the relative position of the furnace at each stage. (c) Relative mass loss as a function of temperature for NaCl, MoO_3 and the mixture of MoO_3+NaCl, measured my thermogravimetric analysis. These measurements were recorded in a Q600-TA instrument under a constant heating rate of 10 °Cmin^{-1} under Ar flow.

Electrochemical characterization set-up

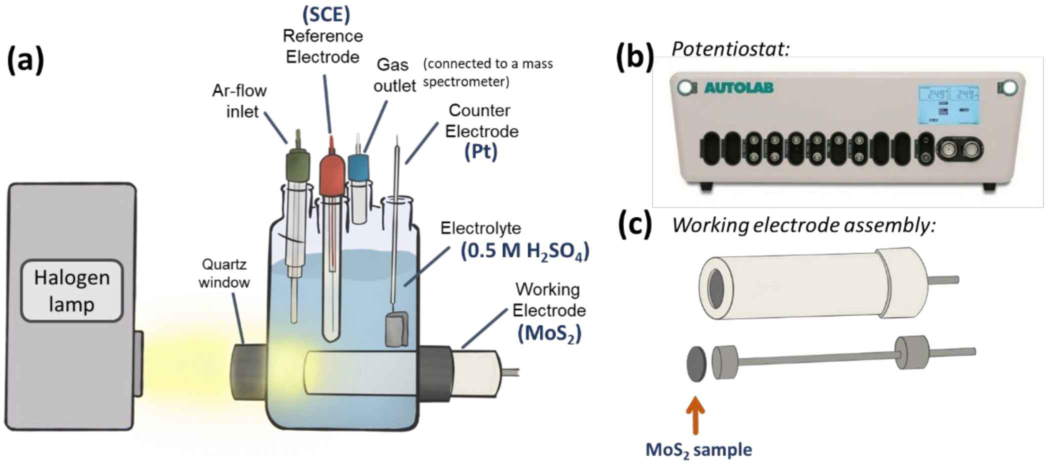

Figure S2. (a) Schematic of the photoelectrochemical set-up. (b) Photograph of the potentiostat employed for the electrochemical measurements. (c) Schematic of the working electrode assembly. The sample is in contact with a metallic connection, which is embedded inside a Teflon cover.

Results

1. *Growth of homogeneous nanocrystalline MoS_2 ultrathin layers*

1.1.Morphological characterization of the MoS_2 samples

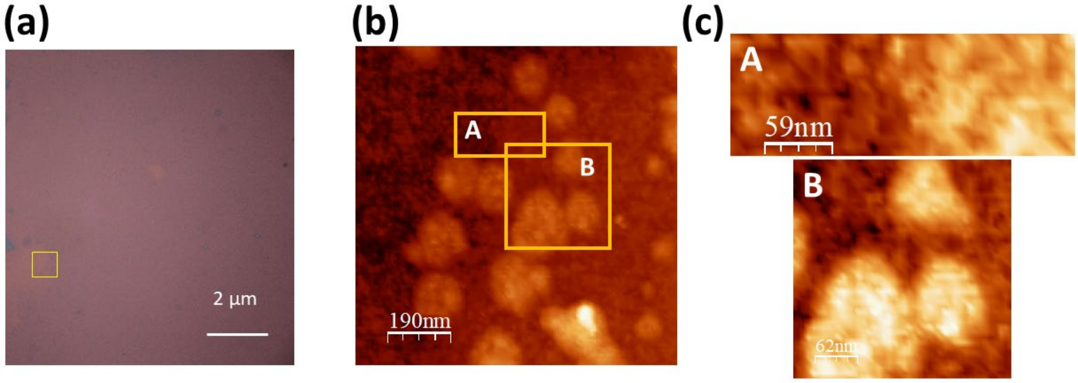

Figure S3. (a) Optical microscopy image of a MoS_2 layer grown onto Si/SiO_2 substrate. Slight changes in the thickness of the samples produce a clear contrast in the colour of the optical images when using these substrates. Blue regions in the images correspond to the bare substrate regions, where AFM images were taken to determine the layer thickness (marked with a yellow square). (b) AFM image shown in the main text (Figure 1a) of a MoS_2 layer grown on Si/SiO_2 substrate. (c) Zoom of the two regions indicated in (b).

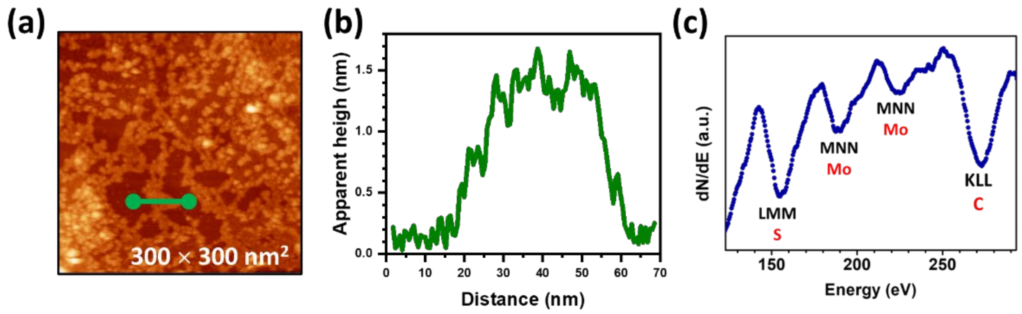

Figure S4. (a) STM image in a 300 x 300 nm² area on a MoS_2 sample grown onto HOPG. The green line indicates the cross-section in which the apparent height has been measured. Tunneling parameters: Vs = 2.8 V; I_T = 12 pA. (b) Apparent height. (c) Coupled with the STM measurements we carried out Auger spectroscopy which revealed the presence of Mo and S in our samples. In addition to these two elements Carbon peaks were observed, which come from the HOPG substrate. Electron beam energy 2.8 keV.

1.2.Optical characterization of the MoS_2 samples

The refractive index of the MoS_2 layers has been obtained from the optical absorption and differential reflectance spectra experimentally measured by using the following relationship [1]: $\frac{\Delta R}{R_0} = \frac{4n\alpha d}{n_s^2-1}$; where n and n_s are the refractive indexes of the MoS_2 and the silica substrate, respectively, α is the optical absorption coefficient and d is the thickness of the MoS_2 layer. There is a qualitatively good agreement with previously published data for the refractive index of MoS_2 [2]. However, previous reported data are from micrometric-sized MoS_2 flakes, whereas our layers are formed by nanometric sizes. Therefore, a size effect on the refractive index of MoS_2 cannot be excluded.

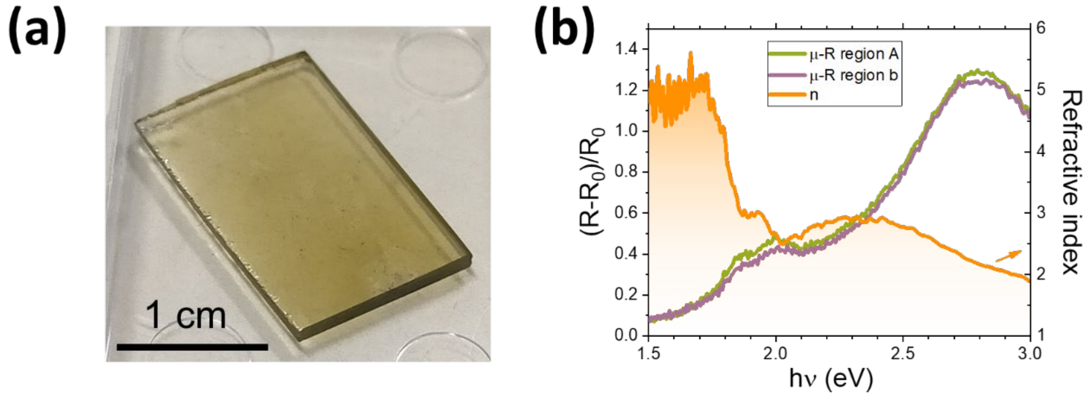

Figure S5. (a) Picture of a MoS_2 sample grown on fused silica in which the high homogeneity of our samples can be appreciated. (b) Differential reflectance measurements acquired with a micro-reflectance setup obtained in two different zones of the sample and the refractive index obtained from the optical density (see Figure 2c in the manuscript) and the differential reflectance.

1.3. Raman characterization of the MoS_2 samples

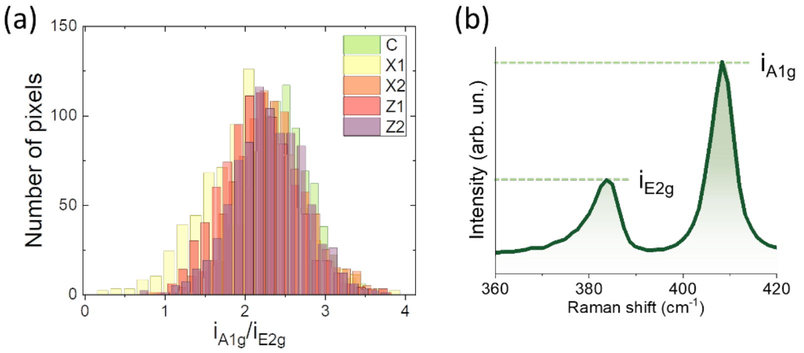

Figure S6. (a) Histogram of the relative intensities between A_{1g} and E_{2g} Raman bands for the five regions analysed in Figure 3a. (b) Representative Raman spectrum in which the intensities of A_{1g} and E_{2g} are indicated.

1.4. XPS characterization of the MoS_2 samples

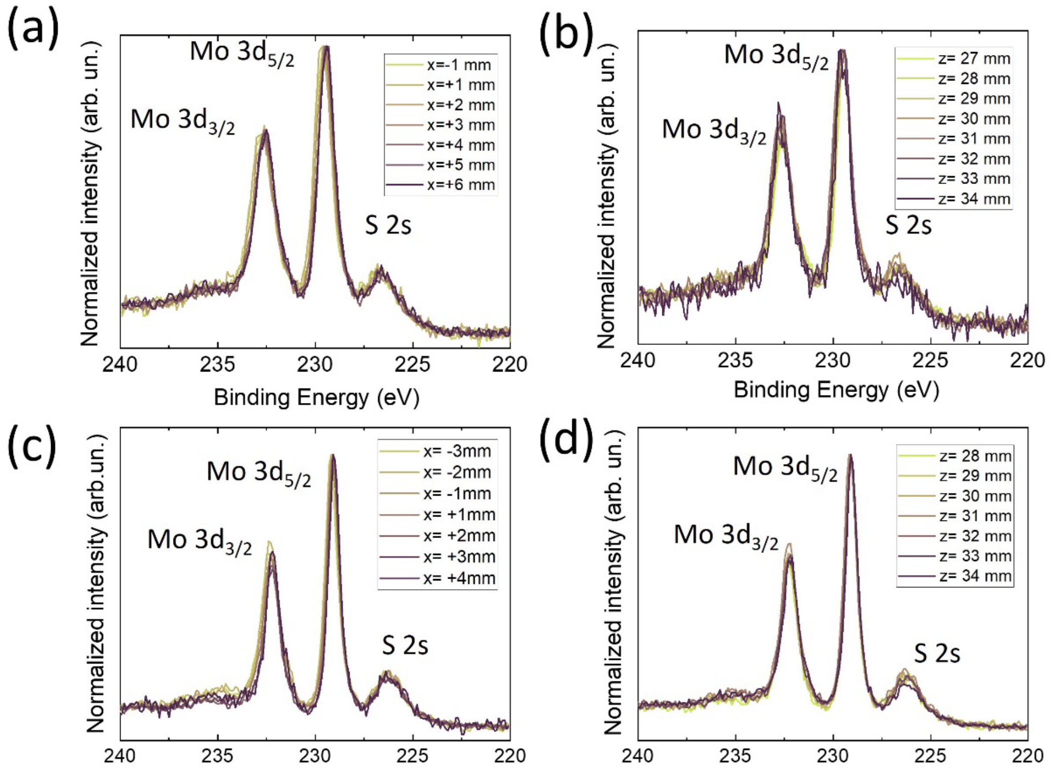

Figure S7. XPS spatial line scans recorded across two perpendicular directions in two different samples. Sample MS-1.7 (a) x-direction, (b) z- direction, and sample MS-2.1 (c) x-direction, (d) z- direction. Scans have been done with a step of 1 mm and a spot size in the 0.1 mm range. The region of the Mo 3d and S 2s peaks have been selected to characterize the chemical bonding state and the stoichiometry. Spectra have been background subtracted and normalized to the most intense peak for a better comparison. The variation in the peak position and the relative intensities is negligible, confirming the chemical homogeneity along the samples.

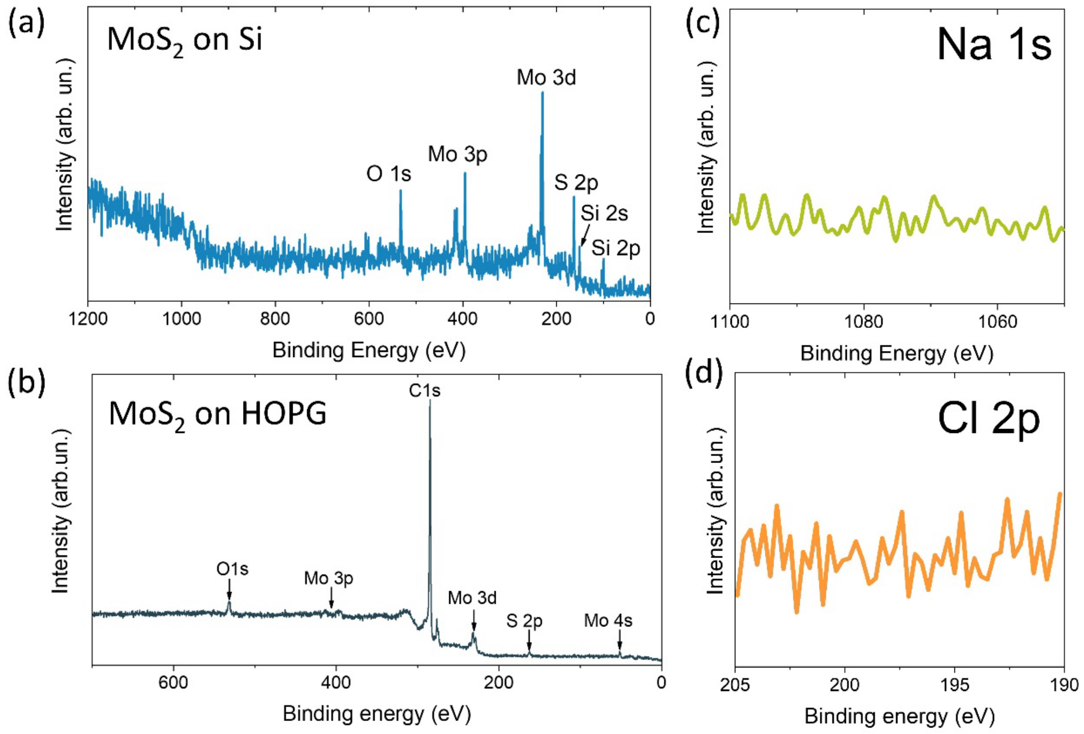

Figure S8. XPS survey spectra for MoS_2 samples grown over a Si substrate (a) and over a HOPG substrate (b) acquired with different experimental setups. Main peaks are identified. Zoom to the Na 1s (c) and Cl 2p regions. There are not any signals of Na nor Cl residuals in either spectrum.

2. *Tuning the sulfur content in the MoS_2 layers*

Table S1. Binding energy for S 2p and Mo 3p components of the XPS spectra compared to literature. Representative spectra in these two regions are shown in Figure S8 for MS-2.1.

	Binding energy (eV)					
	**S^{2-} 2p$_{3/2}$**	**S^{2-} 2p$_{1/2}$**	**Mo^{4+} 3p$_{3/2}$**	**Mo^{4+} 3p$_{1/2}$**	**Mo^{6+} 3p$_{3/2}$**	**Mo^{6+} 3p$^{1/2}$**
**MS-2.3**	162.2 ±0.1	163.4±0.1	395.7±0.1	413.2±0.1	398.0±0.2	416.3±0.1
**MS-2.1**	162.1 ±0.1	163.3±0.1	395.6±0.1	413.1±0.1	397.5±0.1	416.2±0.2
**MS-1.7**	162.2 ±0.1	163.4±0.1	395.5±0.1	413.1±0.1	397.7±0.1	417.2±0.2
**[3]**	162.94	Not resolved	395.43	413.02		
**[4]**	162.0	163.2	395.0	412.3		
**[5]**	162.6	163.8	395.0	412.3		
**[6]**	161.9	163.1	Not resolved	Not resolved		

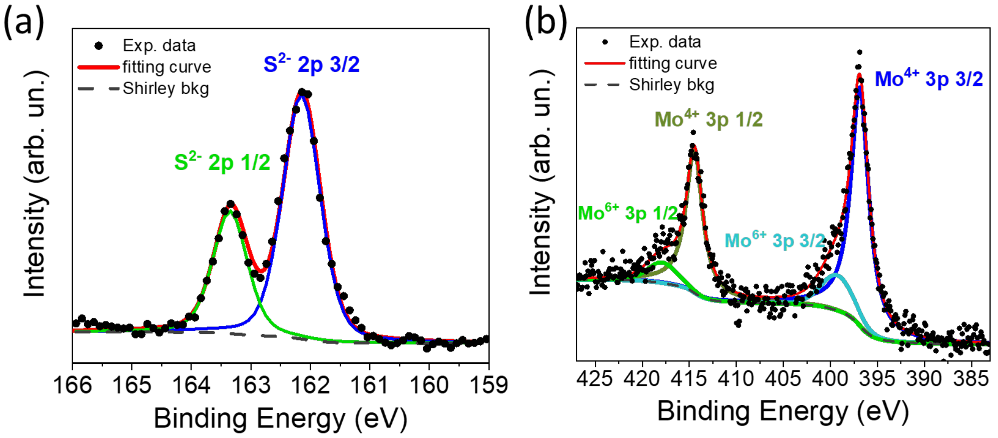

Figure S9. XPS spectrum of a MoS_2 sample (MS-2.1) (a) in the S 2p region and (b) in the Mo 3p region. Both spectra recorded at a pass energy of 5 eV.

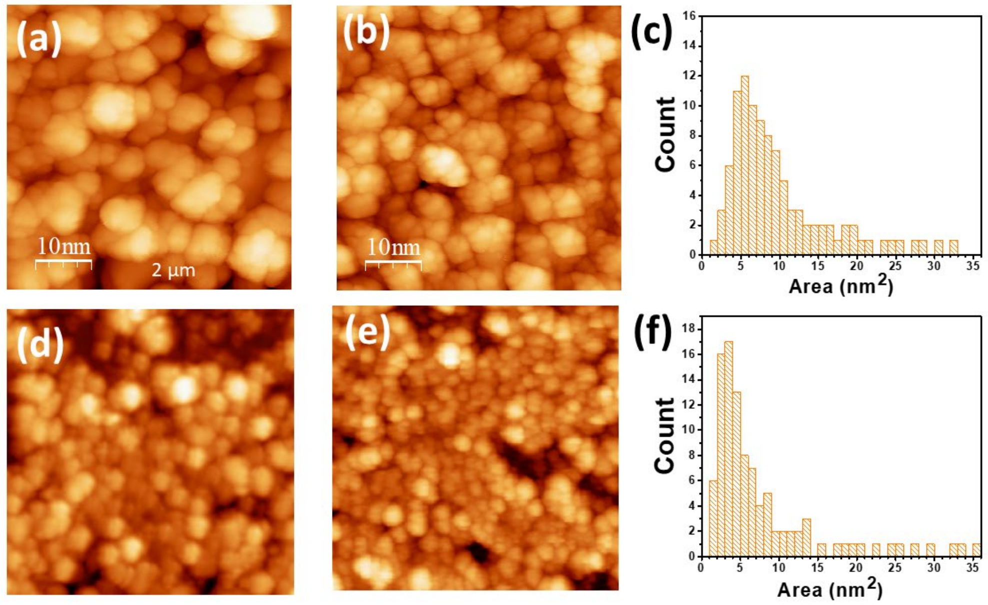

Figure S10. (a) STM images acquired on MoS_2 samples grown onto HOPG under different experimental conditions: (a) and (b) in a sample grown without the use of a H_2 flow (Ar flow 150 sccm), with tunneling parameters: Vs = -2.5 V; I_T = 20 pA in (a) and Vs = -2.5 V; I_T = 9 pA in (b); (d) and (e) in a sample grown with a H_2 flow (Ar flow 150 sccm+ H2 flow of 30 sccm), with tunneling parameters: Vs = 2.73 V; I_T = 60 pA in (d) and Vs = 2.80 V; I_T = 12 pA in (e); All images correspond to an area of 50 x 50 nm^2. The statistical analysis of the size distribution of the MoS_2 nanocrystals for samples grown without and with a H_2 flow are shown in (c) and (f), respectively.

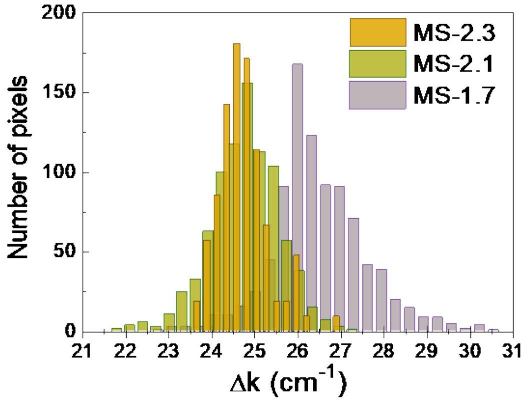

Figure S11. Histogram distributions of Δk values in the Raman spectra for three samples with different S/Mo ratio (2.3, 2.1 and 1.7)

3. *Use of MoS_2 layers as electrocatalysts for the Hydrogen Evolution Reaction*

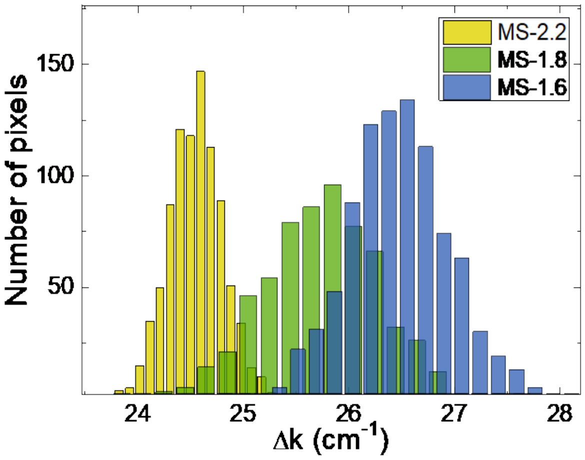

Figure S12. Histogram distributions of Δk values in the Raman spectra for three samples with different S/Mo ratio measured in electrochemistry (2.2, 1.8 and 1.6).

Table S2. Values of the fitting parameters obtained from the Tafel plots shown in Figure 5b in the main text. $\eta = a.\log|i| + b$

Sample	a (mV/dec)	b (V)
GC	295 ± 1	1.08 ± 0.3
MS2-2.2	166.4 ± 0.4	0.4972 ± 0.0002
MS2-1.8	126.2 ± 0.3	0.2667 ± 0.0002
MS2-1.6	147± 1	0.4865 ± 0.0004

To evaluate the possible influence of Pt leaching and deposition onto our electrodes, that could obscure our electrochemical results, we have performed some additional characterizations. We were unable to perform XPS characterizations of our MoS_2 electrodes to evaluate the possible contamination with Pt because these samples did not fit into our sample holders for XPS characterizations. However, we determined the concentration of Pt in the electrolyte before and after long electrolysis times (about 20 h) by ICP-MS. These analyses were done with the electrolytes used with different samples and showed a slight increase in Pt concentration after electrolysis, in the range of a 2-5 µg/l. In addition to the low concentration values of Pt found in the electrolytes, it must be noticed that our electrodes showed an excellent stability in the chronoamperometry curves over long time test (over 100 h), as shown in Figure 5e of the manuscript. This observation supports that Pt deposition has a weak effect on our electrochemical results.

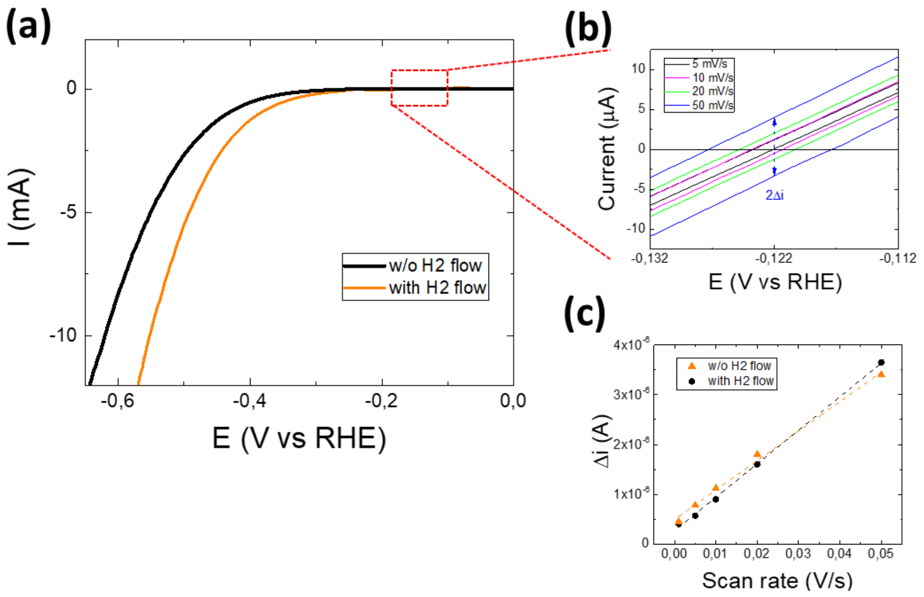

Figure S13. (a) LSV curves recorded with MoS_2 electrodes grown on GC substrates with and without a H_2 flow in CVD. Both curves have been recorded after 20 h of chronoamperometric measurements at the same electrode potential (-0.45 V vs RHE). In the sample obtained without a H_2 flow a Pt CE was used, whereas the sample prepared with a H_2 flow (150 sccm Ar+ 30 sccm H_2) was measured using a Ti CE. (b) CV curves recorded at different scan rates with the sample prepared using a H_2 flow. These curves have been measured in potential window where no electrochemical reactions take place (marked with a red rectangle in (a)). Therefore, the recorded electrochemical currents are ascribed to the charge and discharge of the electrochemical double layer. (c) Dependence of the Δi values defined in (b) on the applied scan rate. The slope of these plots allows the determination of the electrochemical surface active area.

Additional electrochemical experiments were performed by using Ti counter electrodes instead of Pt ones. Figure S13 shows the comparison of the LSV curves recorded with a sample prepared without the use of a H_2 flow (therefore having a low content of S-vacancies) and with another sample prepared using a H_2 flow (having a higher content of S-vacancies). Both curves have been recorded after 20 h of chronoamperometric measurements at the same electrode potential (-0.45 V vs RHE). Both in the long time amperometry records and in the LSV curves, we used a Pt CE with the sample obtained without a H_2 flow, whereas a Ti CE was used with in the sample prepared with a H_2 flow. According to the LSV curves shown in Figure S13, the sample containing a higher number of S-vacancies (the one prepared under Ar+H_2 mixtures) shows better electrocatalytic activity than the one prepared under pure Ar flow. This demonstrates that the improved behaviour is related to the characteristics of the samples and is not an experimental artifact related to the use of a Pt or Ti CE.

On the other hand, electrochemical surface active area (ESCA) of electrodes prepared under different CVD conditions (thus having different electrocatalytic activities) have been measured in order to ascertain if the observed activities are related to the higher surface area of the electrodes. We have used the method reported in reference [7] and the obtained results are shown in Figures S13 (b) and (c). Double layer capacitances of 59±4 μF and 67±2 μF have been obtained for the sample prepared without a H_2 flow and for the one prepared with a H_2 flow, respectively. By considering a typical value of the electrochemical double layer capacitance of 40 μF/cm^2 [7], the real surface areas of the electrodes are of about 1.4-1.7 cm^2. These values are close to the apparent surface areas (0.8 cm^2). This implies that in spite the different size of the MoS_2 nanocrystals obtained with different CVD conditions (see Figure S10), their surfaces are quite flat and present a low roughness in both cases.

Raman analyses indicate that the electrodes are similar before and after the electrochemical tests (sample MS-1.6).

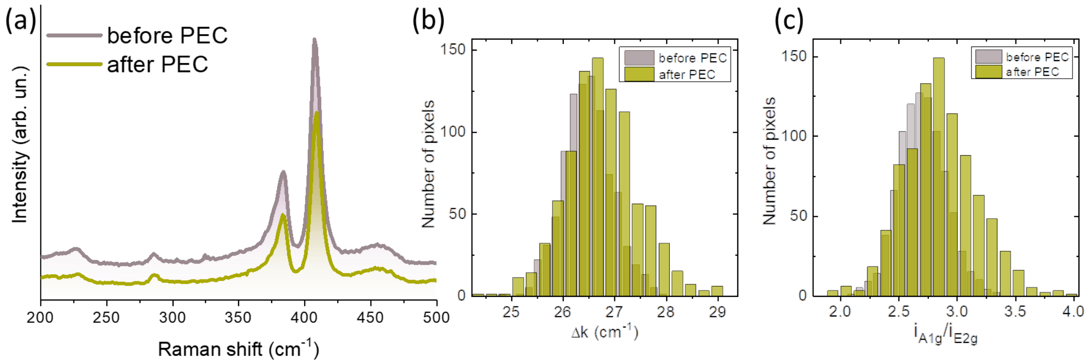

Figure S14. (a) Raman spectra before and after the PEC measurements for sample MS-1.6. Histogram distributions of (b) Δk values and (c) A1g/E2g intensity ratio before and after the PEC measurements.

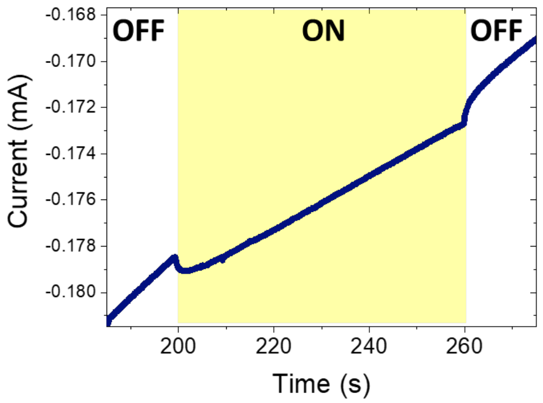

Figure S15. Chronoamperometry measurement at dark (OFF) and under illumination (ON) conditions for sample MS-2.2 at -0.38V vs RHE.

References

[1] Mcintyre, J. D. E.; Aspnes, D. E. Differential Reflection Spectroscopy of Very Thin Surface Films. *Surface Science* **1971**, *24*, 417-434.

[2] Zhang, H.; Ma, Y.; Wan, Y.; Rong, X.; Xie, Z.; Wang, W.; Dai, L. Measuring the Refractive Index of Highly Crystalline Monolayer MoS2 with High Confidence. *Sci. Rep.* **2015,** *5,* 8840.

[3] Turner, N. H.; Singlet, A. M. Determination of Peak Positions and Areas from Wide-Scan XPS Spectra. *Surf. Int. Anal.* **1990,** *15,* 215-222.

[4] Ganta, D.; Sinha, S.; Haasch, R. T. 2-D Material Molybdenum Disulfide Analyzed by XPS. *Surface Science Spectra* **2014,** *21,* 19–27.

[5] Qiu, D.; Lee, D. U.; Pak, S. W.; Kim, E. K. Structural and Optical Properties of MoS_2 Layers Grown by Successive Two-Step Chemical Vapor Deposition Method. *Thin Solid Films* **2015,** *587,* 47–51.

[6] Hussain, S.; Vikraman, D.; Singh, A. K.; Iqbal, M. Z.; Khan, M. F.; Kumar, P.; Choi, D. C.; Song, W.; An, K. S.; Eom, J.; Lee, W. G.; Jung, J. Large-area, Continuous and High Electrical Performances of Bilayer to Few Layers MoS_2 Fabricated by RF Sputtering via Post-Deposition Annealing Method. *Sci. Rep*. **2016,** *6,* 30791.

[7] Connor, P.; Schuch, J.; Kaiser, B.; Jaegermann, W. The Determination of Electrochemical Active Surface Area and Specific Capacity Revisited for the System MnOx as an Oxygen Evolution Catalyst. *Zeitschrift für Physikalische Chemie* **2020,** *234,* 979-994.
